# Antimicrobial use and antimicrobial susceptibility in *Escherichia coli* on small- and medium-scale pig farms in north-eastern Thailand

**DOI:** 10.1186/s13756-017-0233-9

**Published:** 2017-07-17

**Authors:** G. Ström, M. Halje, D. Karlsson, J. Jiwakanon, M. Pringle, L.-L. Fernström, U. Magnusson

**Affiliations:** 1 Department of Clinical Sciences, Swedish University of Agricultural Sciences, Uppsala, Sweden; 2 Research Group for Preventive Technology in Livestock, Faculty of Veterinary Medicine, Khon Kaen University, Khon Kaen, Thailand; 3 National Veterinary Institute, Uppsala, Sweden; 4 Department of Biomedical Sciences and Veterinary Public Health, Swedish University of Agricultural Sciences, Uppsala, Sweden

**Keywords:** Antimicrobial use, Antimicrobial resistance, *E. coli*, Pig production, Farm size, Thailand

## Abstract

**Background:**

Intensification of livestock production seen in many low- and middle-income countries is often believed to be associated with increased use of antimicrobials, and may hence contribute to the emergence of antimicrobial resistance. The aim of this study was to map antimicrobial use on small- (*n* = 25) and medium-scale (*n* = 27) pig farms in north-eastern Thailand, and to compare antimicrobial susceptibility of commensal *Escherichia coli* isolated from sows on these farms.

**Methods:**

Information regarding pig husbandry and antimicrobial treatment regimens was obtained by the use of semi-structured questionnaires. Faecal samples were collected from three healthy sows at each farm, and *Escherichia coli* was cultured and analysed for antimicrobial susceptibility using the broth microdilution method. Multilevel regression models were used to compare antimicrobial susceptibility between isolates from small- and medium-scale farms.

**Results:**

All farms included in the study administered antimicrobials to their sows. Small-scale farmers most commonly (64%) decided themselves when to give antimicrobials and the majority (60%) bought the medicines at the local store or pharmacy, whereas farmers on medium-scale farms always discussed antimicrobial treatment with a veterinarian. Medium-scale farms used a greater diversity of antimicrobials than small-scale farms and did also administer antimicrobials in feed to a higher extent. High levels of antimicrobial resistance to several critically important antimicrobials for human medicine (including ciprofloxacin, streptomycin and ampicillin) were found in isolates from both small- and medium-scale farms. Resistance levels were significantly (*P* < 0.05) higher in isolates from medium-scale farms for several of the antimicrobials tested, as well as the level of multidrug-resistance (*P* = 0.026).

**Conclusion:**

The routines regarding access and administration of antimicrobials differed between the small- and medium-scale farms. Although the level of antimicrobial resistance, as well as multidrug-resistance, was higher in isolates from medium-scale farms, it cannot be concluded if this increase is a consequence of a more abundant use of antimicrobials, or a result of differences in administration routines.

## Background

The emergence of antimicrobial resistance (AMR) has become a serious global health concern, and imprudent use of antimicrobials has been recognized as a contributing factor in the selection for resistant bacterial populations [1]. Although much of the resistance seen in human medicine originates from inappropriate use in humans [2], antimicrobial use within the livestock sector is believed to contribute to AMR through increased selective pressure and through generating resistance reservoirs [3, 4].

The level of AMR has been shown to be highest in low- and middle-income countries, particularly those in South and Southeast Asia [5]. In Thailand, infections with antimicrobial resistant bacteria in humans have been estimated to be the cause of almost 90,000 hospitalisations and 38,000 mortalities each year [6], and high levels of resistance have been documented in the environment and along the food production chain [7].

As *Escherichia coli* are intestinal commensal bacteria present in both animals and humans, and therefore subjected to a high selection pressure driven by the antimicrobials to which their hosts are exposed, they may serve as a good indicator to monitor the general level of AMR in a human or livestock population [8]. Also, although commensal bacteria are normally harmless, they may constitute a reservoir of resistance genes that may be transmitted to pathogenic bacteria [9].

Today’s intensive livestock production often depends on antimicrobials to maintain the health and productivity of the livestock [10]. Antimicrobials are not only used to treat infectious diseases, but are also sometimes used sub-therapeutically for growth promotion, a practice associated with a high potential for selection of resistant pathogens [11]. Intensification of livestock production in many low- and middle-income countries, as exemplified by Thailand’s pig sector [12, 13], is often believed to be driving an increased use of antimicrobials and thereby also an increased emergence of AMR [10, 14]. Here we explored the validity of that assertion in a country with an expanding pig sector. We therefore investigated antimicrobial use on small- and medium-scale pig farms in north-eastern Thailand, and compared antimicrobial susceptibility of commensal *E. coli* isolated from sows on these farms.

## Methods

### Study area

This cross-sectional study was conducted on small- and medium-scale pig farms situated near the city Khon Kaen in north-eastern Thailand. In 2013, the country produced more than 16 million pigs for slaughter [12]. Among the 220,000 households that raised pigs, 94% raised less than 50 pigs per year. On the other hand, two large companies owned about 46% of all breeder pigs. Currently, there are about 200 such contractual, company-owned pig farms in the Khon Kaen province and the density of the total number of pigs, as well as small - scale farms (less than 50 heads per farm), are about the averages for the provinces in Thailand [13]. In the last decade, Thailand’s pig sector has intensified and farm sizes have increased; a trend that is expected to continue [13].

### Study design and data collection

The study was conducted between September and November 2015. Inclusion criteria for small-scale farms were that the farm kept sows for breeding, with a maximum of 20 sows, whereas medium-scale farms were defined as farms with between 100 and 500 sows, a classification relating to previous work in Thailand where such farms have been named “smallholders” and “small large-scale farms”, respectively [13]. In order to make a distinct difference in size between the small- and medium-scale farms in the present study, no farms with 21–99 sows were included. From a governmental list of villages with pig farm(s), the heads in randomly selected villages were contacted for checking that there were pig farms at the time of the fieldwork. The villages with positive responses were visited and all farms where the farmers were at home agreed to participate. For the medium-scale farms, that all but one belonged to two companies, the company officers were asked to make a choice of farms to be equally distributed over the province. By this procedure, 25 small-scale and 27 medium-scale farms were included in the study.

A faecal sample was collected, using a rectal swab, from the individual pen of three randomly chosen healthy sows at each farm and added to sterile plastic tubes containing Amies medium. At the small-scale farms, sampling was performed by the authors (MH, DK), and at the medium-scale farms by local veterinary assistants who were thoroughly instructed beforehand how to collect the samples in a correct manner. Sampling was performed at mid-day and samples were transported to Khon Kaen University within 1–6 h and stored at 2-8 °C until analysis, which was performed within 48 h after sampling. Because not all small-scale farms had three healthy sows, only 69 samples were obtained from these farms.

A semi-structured questionnaire, with questions on farm characteristics, pig husbandry and routines for antimicrobial treatment, was administered to the person responsible for the pigs at each farm. The questionnaire was in English and had been pre-tested on pig farms not involved in the subsequent study, after which necessary adjustments were made. Interviews were performed by the authors MH and DK together with a Thai speaking veterinary student at the Khon Kaen University who translated the questions to Thai and the farmer’s answers to English. The interviews took about 40 min each.

### Antimicrobial susceptibility testing

Each faecal sample was streaked on MacConkey agar and incubated at 44 °C overnight. Suspected *E. coli* isolates were sub-cultured on blood agar, incubated at 37 °C overnight and tested for production of tryptophanase (indole). One indole positive isolate was selected from each faecal sample and further tested for antimicrobial susceptibility.

Susceptibility testing was performed by broth microdilution, using the growth method inoculum preparation, according to the standards described by the Clinical and Laboratory Standards Institute (CLSI) [15]. Microdilution panels (VetMIC GN-mo, National Veterinary Institute, Uppsala, Sweden) were used to determine susceptibility to 13 antimicrobials (ciprofloxacin, nalidixid acid, gentamicin, streptomycin, tetracycline, florfenicol, chloramphenicol, colistin, sulfamethoxazole, trimethoprim, ampicillin, cefotaxime, ceftazidime). *Escherichia. coli* ATCC 25922 was used as quality control strain.

The Minimum Inhibitory Concentration (MIC) for each antimicrobial was recorded and epidemiological cut-off values (ECOFFs), defined by the European Committee on Antimicrobial Susceptibility Testing [16], were used to differentiate between wild-type and non-wild-type isolates. Wild-type and non-wild-type isolates will henceforth be referred to as susceptible and resistant, respectively. Multidrug-resistance was defined as isolates resistant to at least three different categories (i.e. with different mechanisms of action) of the antimicrobials tested [17].

### Statistical analyses

Statistical analyses were conducted in SAS software 9.4 (SAS Institute Inc., Cary, NC). Descriptive statistics were computed to define farm characteristics. To investigate associations between management factors, such as use of antimicrobials and veterinary services, and resistance against different types of antimicrobials, univariable logistic regression and Fisher’s exact test were used where applicable. Multilevel regression models were used to compare susceptibility patterns between the small- and medium-scale farms. As isolates from the same farm were assumed to be more alike, generalised estimating equations (GEE) were used to account for the effect of clustering within farms. The statistical significance level was defined as a two-tailed *P*-value <0.05.

## Results

### Farm characteristics

At the small-scale farms, all privately owned, the number of sows varied from 1 to 19, with a median of 5 sows (25^th^ and 95^th^ percentiles: 3 and 12 sows). Most of these farms (60%) practised a ‘farrow-to-finish’ system, where pigs were raised from birth to slaughter. The rest of the small-scale farms were either breeding farms (28%), where the piglets were sold to other farmers after weaning, or farms that practised a combination of the two systems (12%).

The medium-scale farms in this study, except for one farm that was privately owned and managed, were contract farms contracted by two different companies. The number of sows ranged from 100 to 400, with a median number of 210 sows (25^th^ and 95^th^ percentiles: 155 and 327 sows). All medium-scale farms were breeding farms, and the privately managed farm also combined this with a ‘farrow-to-finish’ system.

None of the farms in this study kept their pigs freely roaming; instead the pigs were kept in enclosures, such as concrete pens or metal crates.

### Antimicrobial use

All farms in this study administered antimicrobials to their sows, though only 21 of the 25 small-scale farms knew the names of the antimicrobials used. Small-scale farmers most commonly decided themselves when to give antimicrobials and the majority bought the drugs at the local store or pharmacy (Table 1). On all medium-scale farms, the farmer discussed with a veterinarian when to give antimicrobials to the animals, and the contract farms received the antimicrobials through their respective contracting company. The privately managed medium-scale farm received antimicrobials through a pharmaceutical company. Furthermore, antimicrobials were added to the feed on all medium-scale farms, a practice that was performed on only one of the small-scale farms.

**Table 1:** Practices related to use and access to antimicrobials on the small- and medium-scale pig farms

	Small-scale % (*n* = 25)	Medium-scale % (*n* = 27)
Antimicrobials injected for disease treatment	100 (25)	100 (27)
Antimicrobials added to, or included in, feed^a^	4 (1)	100 (27)
Person who decides on when to give antimicrobials		
Farmer	68 (17)	0 (0)
Veterinarian	32 (8)	100 (27)
Source of antimicrobials		
From veterinarian	32 (8)	0 (0)
From local store/pharmacy	60 (15)	0 (0)
From the contracting company	4 (1)	96 (26)
Other	4 (1)	4 (1)

^a^On regular basis

The number and types of antimicrobials used differed between small- and medium-scale farms (Table 2). Penicillin and streptomycin were used as injectable drugs on all the medium-scale farms, compared to only 19% of the small-scale farms. Small-scale farms most commonly used amoxicillin (38%) and enrofloxacin (57%) as injectable drugs. For the contract farms, the two contracting companies had different regimes regarding treatment with antimicrobials. Both companies provided penicillin and streptomycin as injectable drugs to their contract farms, and one of the companies also provided enrofloxacin. The companies either provided kitasamycin or oxytetracycline as in-feed antimicrobials to their respective farms. On the privately managed medium-scale farm, penicillin, streptomycin, amoxicillin, oxytetracycline and cefotaxime were administered by injection, and amoxicillin and colistin were provided with the feed. Unfortunately, no information regarding the amount of antimicrobials used could be obtained in this study.

**Table 2:** Antimicrobials administered to sows on the small- (*n* = 21^a^) and medium-scale farms (*n* = 27)

Antimicrobials	Small-scale^a^																					Medium-scale																										
*Injection*																																																
Penicillin G^b^											●	●				●	●	●				●	●	●	●	●	●	●	●	●	●	●	●	●	●	●	●	●	●	●	●	●	●	●	●	●	●	●
Streptomycin^b^											●	●				●	●	●				●	●	●	●	●	●	●	●	●	●	●	●	●	●	●	●	●	●	●	●	●	●	●	●	●	●	●
Amoxicillin^b^				●			●			●		●	●		●			●			●																											●
Oxytetracycline													●																																			●
Cefotaxime^b^																																																●
Enrofloxacin		●			●	●		●	●			●	●		●	●	●	●		●																●	●	●	●	●	●	●	●	●	●	●	●	
Kanamycin^b^	●	●	●									●							●																													
Florfenicol																																																
Gentamicin^b^														●																																		
*In feed*																																																
Amoxicillin^b^		●																																														●
Colistin^b^																																																●
Kitasamycin																						●	●	●	●	●	●	●	●	●	●	●	●	●	●													
Oxytetracycline		●																																		●	●	●	●	●	●	●	●	●	●	●	●	
Sulfonamide		●																																														
Farm no	1	2	3	4	5	6	7	8	9	10	11	12	13	14	15	16	17	18	19	20	21	26	27	28	29	30	31	32	33	34	35	36	37	38	39	40	41	42	43	44	45	46	47	48	49	50	51	52

^a^From four of the small-scale farms, information on which antimicrobials that were used was not obtained and these farms are thus not displayed in the figure

^b^Antimicrobial considered to be critically important for human medicine according to WHO [22]

### Antimicrobial susceptibility

In total, 69 and 81 isolates were obtained from the small- and medium-scale farms, respectively. Around 90% (62/69) of isolates from the small-scale farms showed resistance to at least one category of antimicrobials. The corresponding figure for medium-scale farms was 98% (79/81). The MIC distributions for isolates from the small- and medium-scale farms are presented in Tables 3 and 4, respectively.

**Table 3:**
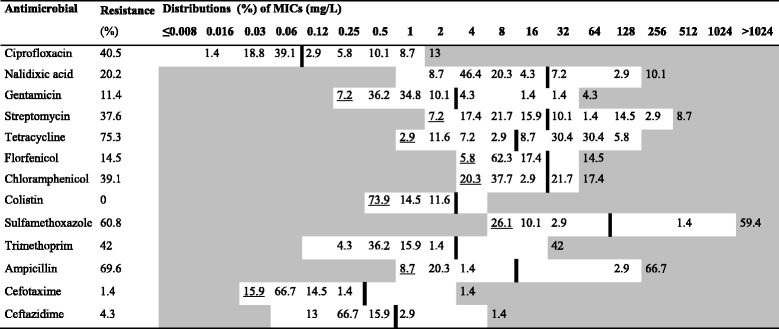
Resistance and distributions of Minimum Inhibitory Concentrations (MIC) for *E. coli* isolates from small-scale farms

White fields denote range of dilutions tested for each substance. MICs higher than the highest concentration tested are given as the concentration closest above the range. MICs equal to or lower than the lowest concentration tested are presented underlined. The ECOFF [16] for each substance is presented as a vertical line

**Table 4:**
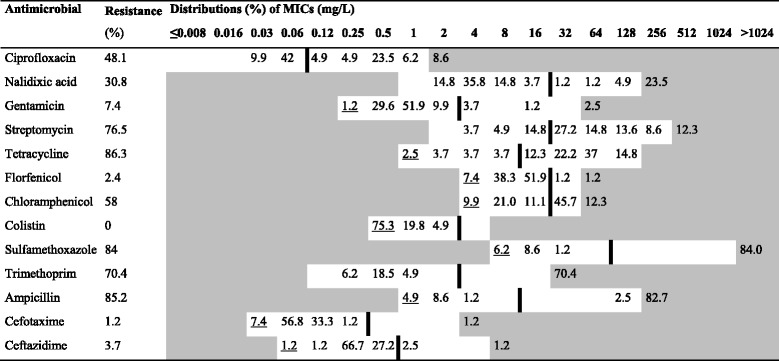
Resistance and distributions of Minimum Inhibitory Concentrations (MIC) for *E. coli* isolates from medium-scale farms

White fields denote range of dilutions tested for each substance. MICs higher than the highest concentration tested are given as the concentration closest above the range. MICs equal to or lower than the lowest concentration tested are presented underlined. The ECOFF [16] for each substance is presented as a vertical line

For both small- and medium-scale farms, most isolates showed resistance to ampicillin, tetracycline and sulfamethoxazole, ranging from 61% to 86% (for details see Table 5). No isolates showed resistance to colistin, and only a few isolates were resistant to the third generation cephalosporins cefotaxime and ceftazidime. Isolates obtained from medium-scale farms were significantly more likely to be resistant to streptomycin (*P* < 0.001), sulfamethoxazole (*P* = 0.006), trimethoprim (*P* < 0.001) and chloramphenicol (*P* = 0.023), compared to isolates from small-scale farms. Multidrug-resistance was more common (*P* = 0.026) in isolates from medium-scale farms (89%), than in isolates from small-scale farms (74%).

**Table 5:** Antimicrobial resistance parameters of *E. coli* isolates from pigs on the small- and medium-scale farms

Antimicrobial	Resistant isolates % (n)		*P*-value	OR (95% CI)^a^
	Small-scale (*n* = 69)	Medium-scale (*n* = 81)		
Ciprofloxacin^†^	41 (28)	48 (39)	0.54	
Nalidixic acid^†^	20 (14)	31 (25)	0.21	
Gentamicin^†^	11 (8)	7 (6)	0.39	
Streptomycin^†^	38 (26)	77 (62)	<0.001	5.4 (2.7-11)
Tetracycline	75 (52)	86 (70)	0.09	2.1 (0.9-4.8)
Florfenicol	14 (10)	2 (2)	0.01	0.1 (0.03-0.7)
Chloramphenicol	39 (27)	58 (47)	0.02	2.2 (1.1-4.1)
Colistin^†^	0 (0)	0 (0)	-	-
Sulfamethoxazole	61 (42)	84 (68)	0.002	3.4 (1.6-7.2)
Trimethoprim	42 (29)	70 (57)	<0.001	3.3 (1.7-6.4)
Ampicillin^†^	70 (48)	85 (69)	0.02	2.5 (1.1-5.6)
Cefotaxime^†^	1 (1)	1 (1)	0.9	
Ceftazidime^†^	4 (3)	4 (3)	0.8	
Multidrug-resistance^b^	74 (51)	89 (72)	0.002	3.7 (1.2-8.8)

^†^Antimicrobial classified as critically important for human medicine according to WHO [22]

^a^For the OR estimates, the small-scale farms were used as reference variable and GEE models were used to account for clustering of isolates within farms

^b^Multidrug-resistance defined as an isolate resistant against at least three different categories of the antimicrobial agents tested

Isolates obtained from farms that used enrofloxacin were more likely to show resistance to ciprofloxacin (*P* = 0.02), but not to nalidixic acid (*P* = 0.47). Moreover, all isolates from medium-scale farms that administered oxytetracycline in the feed were resistant to tetracycline.

## Discussion

The overall aim of this study was to investigate possible differences in antimicrobial use in pigs on small- and medium-scale pig farms in Thailand, a country that is experiencing an intensification of the pig sector, like other countries in the region [13]. Furthermore, we compared antimicrobial susceptibility of isolates of *E. coli* from pigs on these farms. This research question stems from the common assertion that farm intensification, in particular in the pig and poultry sectors, is associated with a more abundant use of antimicrobials, and thereby also with higher levels of antimicrobial resistance (AMR) [10, 14].

The levels of AMR presented here are largely similar to those in previous studies on *E. coli* isolated from animals in Southeast Asia, according to a recent review by Nhung et al. [18]. The highest frequencies of resistance in isolates from both small- and medium-scale farms were found against tetracycline, followed by ampicillin. Multidrug-resistance was significantly more commonly observed in isolates from pigs on medium- compared to small-scale farms. These results are in accordance with a study by Love et al. [19], where herd size was reported to be positively associated with higher rates of multidrug-resistance in *E. coli* isolated on pig farms in northern Thailand. Although the medium-scale farms in the present study routinely used a greater diversity of antimicrobials, we do not have any reliable information regarding the amounts of antimicrobials used on the farms. Therefore, no associations between the amounts of administrated antimicrobials and AMR can be determined here.

Penicillin and streptomycin were used on all medium-scale farms, whereas small-scale farms most commonly used enrofloxacin and amoxicillin. Enrofloxacin was also used on almost half of the medium-scale farms. Although this antimicrobial is not used in human medicine, being a quinolone its use in animals selects for resistance to other quinolones that are used for humans. Here we found a positive association between the use of enrofloxacin on the farm and ciprofloxacin resistance in isolates of *E. coli* from the pigs, which indicates the importance of restricting the use of quinolones in animal agriculture. These results are in accordance with studies conducted on pig and chicken farms in Vietnam [20, 21].

Many of the antimicrobials used by the farms in this study are classified by the World Health Organization (WHO) as critically important for human medicine [22], including amoxicillin, cefotaxime and colistin. The recent discovery of the plasmid mediated *mcr-1* gene in bacteria isolated from pigs [23], a gene that confers resistance to colistin and that may be transferred horizontally between bacteria, has emphasised the importance of colistin as a last-resort antimicrobial that should possibly be restricted to human use. Plasmid-mediated colistin resistance has since been detected in almost all regions of the world [24], including in bacteria isolated from Vietnamese pigs [20, 25]. None of the isolates in the present study, however, were resistant to colistin, and it was only used on one of the study farms.

Data presented in this study indicate that medium-scale farms administer antimicrobials to the feed to a higher extent than small-scale farms. Oral administration of antimicrobials is known to increase AMR in *E. coli* from pigs [26]. A possible explanation to the higher use of antimicrobials in feed on the medium-scale farms in this study could be that these farms experience an increased disease pressure that is associated with larger group sizes and higher densities of animals [27], or simply that these farms were more commercially oriented and therefore pursued better animal performance and higher returns from production. We do not have information whether these antimicrobials were intended to promote growth or to prevent sickness in the animals, therefore these are only speculations. However, Thailand recently, as the first country in the region, banned the inclusion of antimicrobials as growth promotors in animal agriculture [28], which is an important step towards a more prudent use of antimicrobials.

We found a difference between the ways in which small- and medium-scale farms accessed antimicrobials, where the latter always consulted a veterinarian before treating sick animals. This difference, that must be interpreted with caution given the sample size, might however not be resulting from increased farm size, but could merely be a consequence of that the medium-scale farms were bound to the routines of the contracting companies, as this was the case for all but one of the medium-scale farms. The two contracting companies, owning about half each of the medium-scale farms in the current study, are very large companies that have operations all over the country, and one may thus assume that the regimes regarding antimicrobial treatment presented here are representative for many medium-scale farms in Thailand.

In this study the emphasis was on antimicrobial use and susceptibility in commensal *E. coli* collected from sows. Sows were sampled as antimicrobial use and resistance in *E. coli* from sows is of epidemiological importance within the herd, as the sow may serve as a reservoir of resistant bacteria and genetic determinants [29]. Whilst the recommendation by the European Food Safety Authority (EFSA) [30] is that samples should be taken from fattener pigs, the approach adopted here was to ensure sufficient number of pigs to sample at each farm, as small-scale farms might not have enough fatteners at the time of visit.

As already mentioned we found a positive association between enrofloxacin use and ciprofloxacin resistance, both being fluoroquinolones used in veterinary and human medicine, respectively. Our analyses also revealed some associations between the use of certain antimicrobials and AMR. Such associations should be interpreted with caution, especially since this study contained relatively few farms and the fact that we could not obtain reliable information from all farms regarding precise treatment regimes, e.g. duration and dosage. Thus, assessing possible associations between the treatment regime and antimicrobial resistance might fall outside the power of this study.

The data from the current study have been interpreted conservatively given the relatively small sample size. Another limitation to take into account when reading the paper is that the selection of farms might not be regarded as entirely random. The antimicrobial activity testing panel used is designed to detect resistance among *E. coli*, therefore there may be resistance in other bacterial species and to other antimicrobials not tested for in this study.

## Conclusions

Considerable reservoirs of antimicrobial resistance were observed in *E. coli* from pigs on both small- and medium-scale farms in north-eastern Thailand. Higher rates of resistance, including multidrug-resistance, were observed on medium-scale farms, which could be a consequence of the greater diversity of antimicrobials used on these farms, as well as of the higher extent to which antimicrobials were administered in the feed. Although we found differences in how antimicrobials were accessed and administered between the small- and medium-scale farms, it cannot be concluded whether these differences could be responsible for the higher rate of antimicrobial resistance observed on the medium-scale farms.

## Abbreviations

AMR: Antimicrobial resistance
CLSI: Clinical and laboratory standards institute
ECOFF: Epidemiological cut-off value
EFSA: European food safety authority
EUCAST: European Committee on Antimicrobial Susceptibility Testing
GEE: Generalised estimating equations
MIC: Minimum inhibitory concentration
SLU: Swedish University of Agricultural Sciences
WHO: World Health Organization
